# Comparison of brachial and central blood pressures using an oscillometric device with 2 or 6 metre tubing lengths for assessment of central pressure during MRI exam

**DOI:** 10.1186/1532-429X-18-S1-P20

**Published:** 2016-01-27

**Authors:** Sebastian Stroer, Gilles Soulat, Sébastian Tavolaro, Sandrine Millasseau, Hakim Khettab, Pierre Boutouyrie, Stéphane Laurent, Elie Mousseaux

**Affiliations:** 1 grid.414093.bEuropean Hospital Georges Pompidou, Paris, France; 2 grid.10992.330000000121880914PARCC INSERM U970 Paris Descartes University, Paris, France; 3 Pulse Wave Consulting, St Leu, France

## Background

Magnetic resonance imaging (MRI) offers the possibility to measure local and regional indices of aortic function. However calculations of these indices usually require blood pressure (BP) values. Up to now, because of its easier availability, brachial BP was used instead of local aortic pressure. The SphygmoCor Xcel system (AtCor Medical, Australia) estimates aortic pressure non-invasively. It consists in a MRI compatible brachial cuff connected via a hose to a recording unit and computer. The aim of this study was to compare brachial and central BP values given by SphgymoCor Xcel with the standard 2 meters hose and a 6 meters hose more suitable for central BP assessment during MRI.

## Methods

After 5 min rest supine, BP was measured simultaneously on both arms with one 2 m SphgymoCor Xcel and one 6 m SphgymoCor Xcel. Arms were randomly assigned. Tubing were then interchanged (cuffs unchanged) and recordings repeated.

## Results

38 patients were studied (63% men). Seven (18%) were treated for hypertension, 2 (5%) for diabetes and 3 (11%) for dyslipidaemia. Median age was 36.8 years (28.5-58.4). Brachial Systolic BP (SBP), Diastolic BP (DBP), Pulse Pressure (PP), central SBP, DBP PP, augmented pressure (AP) and augmentation index (Aix) from the 2 m and 6 m device were strongly correlated (R^2^=0.96, 0.91, 0.78, 0.97, 0.85, 0.95, 0,96 respectively, p < 0.001 for all.). Bland Altman plots showed no statistical difference between 2 and 6 m for brachial and central SBP, DBP, PP values. However there was a difference between AP and Aix recorded with 2 m and 6 m hose (-2.65 ± 1.5 mmHg, p = 0.043 and -5.25 ± 2.93%, p = 0.038 respectively).

## Conclusions

SphygmoCor Xcel device with a 6 m hose, brachial and central BP shows no statistical difference with the standard 2 m hose, allowing data to be collected during MRI exams. However other parameters using waveform morphology such as AP and Aix are not so reliable.

